# Attenuation of the Pressor Response to Tracheal Intubation in Severe Preeclampsia: Relative Efficacies of Nitroglycerine Infusion, Sublingual Nifedipine, and Intravenous Hydralazine

**DOI:** 10.5812/kowsar.22287523.1782

**Published:** 2011-09-26

**Authors:** Mohammadreza Safavi, Azim Honarmand, Neda Azari

**Affiliations:** 1 Anesthesiology and Critical Care Research Center, Isfahan University of Medical Sciences, Isfahan, Iran

**Keywords:** Preeclampsia, Cesarean section, Nifedipine, Hydralazine, Nitroglycerine

## Abstract

**Background:**

The pressor response to laryngoscopy is known to be exaggerated in patients with severe preeclampsia.

**Objectives:**

The aim of the present study was to compare the efficacies of continuous intravenous (IV) infusion of nitroglycerine, IV hydralazine, or sublingual nifedipine in modifying cardiovascular responses to endotracheal intubation, in women with severe preeclampsia undergoing cesarean delivery under general anesthesia.

**Patients and Methods:**

A total of 120 patients undergoing cesarean delivery were randomly divided into 3 groups, each receiving one of the following drugs before intubation: 5 µg/min nitroglycerine administered by continuous IV infusion (Group NTG, n = 40); a 10-mg capsule of nifedipine deposited sublingually (Group NIF, n = 40); or 5–10 mg hydralazine intravenously (Group H, n = 40). Heart rate (HR), systolic arterial pressure (SAP), diastolic arterial pressure (DAP), and mean arterial pressure (MAP) were simultaneously recorded in the mother at pre-induction, pre-intubation, and at 1, 3, 5, and 10 min after intubation.

**Results:**

In contrast to those in group NIF and group H, the patients in group NTG showed no significant increases in HR, SAP, DAP, or MAP after intubation, compared to baseline. The incidence of hypotension was significantly greater in group NIF than in group H or group NTG [15 (37. 5%) vs. 8 (20%) vs. 5 (12. 5%) respectively, P = 0. 025].

**Conclusions:**

In patients with severe preeclampsia undergoing cesarean delivery, a continuous IV infusion of nitroglycerine was able to attenuate the cardiovascular response to intubation to a greater extent than the use of sublingual nifedipine or IV hydralazine, without significant adverse effects on the newborn.

## 1. Background

The pressor response to laryngoscopy and tracheal intubation is a very important issue in hypertensive pregnant patients, involving an increase in systemic and pulmonary arterial pressures and pulmonary capillary wedge pressure (1-4).

In addition, the stress of laryngoscopy can be associated with an increase in intracranial pressure, with the risk of cerebral hemorrhage and cardiac failure with pulmonary edema. Consequently, there is an increased risk of morbidity and mortality of both mother and baby (5). In order to reduce the occurrence and severity of these hemodynamic complications, many drugs such as hydralazine (6), magnesium sulfate (7), labetalol (8), fentanyl (5), trimethaphan (2), sodium nitroprusside (9), lidocaine (10), nitroglycerin (11, 12), and nifedipine (13) have been used with varying degrees of success. Hydralazine has been the antihypertensive of choice for women with severe hypertension in pregnancy for many years (14, 15). However, no studies have been conducted to evaluate the efficacy of hydralazine in attenuation of cardiovascular changes after laryngoscopy and tracheal intubation. Short-acting sublingual nifedipine, a calcium channel blocker, is another effective antihypertensive agent that is sometimes used to control acute, severe hypertension in women with preeclampsia (16, 17). Kumar et al. (13) showed that sublingual nifedipine is effective in attenuating the hypertensive response to laryngoscopy and intubation in pregnancy-induced hypertension.

Nitroglycerin has also been administered for rapid perioperative treatment of maternal hypertension (11). Longmire and colleagues (11) showed that intravenous nitroglycerin infusion is effective in lowering maternal blood pressure (BP) and in blunting hemodynamic responses to endotracheal intubation in severe preeclampsia. In our previous experience, it seemed that among women with severe preeclampsia who are being managed with controlled extracellular volume expansion and MgSO4 loading and maintenance doses, those who received a continuous infusion of IV nitroglycerine during induction of anesthesia had a faster and more precise reduction in their BP levels than those who received sublingual nifedipine or IV hydralazine. To the best of our knowledge, there are no published studies that compare the efficacy of these 3 drugs for this purpose.

## 2. Objectives

Therefore, to test our hypothesis, we conducted the present study, in which we compare the efficacies of continuous IV infusion of nitroglycerine, intravenous hydralazine, or sublingual nifedipine in blunting the cardiovascular effects of laryngoscopy and tracheal intubation, in women with severe preeclampsia undergoing cesarean delivery under general anesthesia.

## 3. Patients and Methods

After obtaining institutional approval and informed consent, 120 patients with severe preeclampsia and gestational age of more than 20 weeks, who presented for elective or urgent cesarean delivery under general anesthesia, were enrolled in this randomized double-blind study. The patients had contraindications for spinal anesthesia. All patients had BP ≥ 160/110 and met the defining criteria of severe preeclampsia according to the American College of Obstetrics and Gynecology (18). Criteria for exclusion from the study were a history of heart failure diagnosed by a cardiologist, difficult airways, or any contraindication for treatment with the study drugs. A difficult airway was anticipated in the presence of a mouth opening less than 2 fingers, a thyromental distance of less than 6 cm, and a grade of III or more in the modified Mallampati test.

Presuming a 10% rate of maternal hypotension using hydralazine (19) compared with 1. 8% without using hydralazine, as well as an α level of 0. 05 and a β level of 0. 20, we estimated that 120 women (40 per group) would need to enroll to reveal a significant decrease in maternal hypotension using nitroglycerine. Gestational age was estimated from the last menstrual period, uterine size at the first prenatal visit, or early sonography if obtained. Laboratory assessment included serial measurement of liver function tests, complete blood cell count, coagulation profile, and renal function tests. All women received intensive monitoring of BP and of cardiac and general status. Fetal status was evaluated each day using the nonstress test from 24 weeks’ gestation and ultrasound at admission or when necessary. Fetal distress was defined as a nonstress test with a baseline variability of < 5 bpm over 60 min, repeated late decelerations, or both. Specific indications for delivery were: fetal distress, abruptio placentae, decline in renal function, HELLP syndrome, persistent severe headache or visual changes, or epigastric pain. Diagnosis of HELLP syndrome was based on a clinical diagnosis of hypertensive disorder and the following laboratory abnormalities: (1) hemolysis, based on 2 or more of the following: characteristic peripheral blood smear, serum lactic dehydrogenase (LDH) ≥ 600 U/L, total bilirubin ≥ 1. 2 mg/dL, and decreased hemoglobin and hematocrit; (2) elevated liver enzymes (2 or 3), defined as aspartate aminotransferase (AST) ≥ 70 U/L, alanine aminotransferase (ALT) ≥ 50, and LDH ≥ 600 U/L; and (3) low platelet count, defined as ≤ 150,000 platelets/mL. Management of severe preeclampsia included bed rest; to prevent seizures, all women initially received magnesium sulfate as a 4-g intravenous loading dose, followed by 1-g intravenously per hour before delivery, intrapartum, and for 24 h postpartum.

As candidate patients were enrolled in the study, they were randomly allocated to 1 of 3 groups according to the agent to be used for attenuation of the pressor response to laryngoscopy: hydralazine (Group H, n = 40), nitroglycerine (Group NTG, n = 40), or nifedipine (Group NIF, n = 40). Allocation was performed in a double-blind fashion using a sealed envelope technique. Group H received 5–10 mg hydralazine intravenously. The initial dose of hydralazine was 5 mg, with further doses of 10 mg at intervals, according to the protocol recommended by the American College of Obstetrics and Gynecology (18). Group NTG received 5 µg/min (50 µL/min) nitroglycerine, administered by continuous IV infusion. The same dose was administered every 5 min until the therapeutic goal was reached, which was a decrease in systolic blood pressure (SBP) to < 140 mmHg but not < 120 mmHg and a decrease in diastolic blood pressure (DBP) to < 100 mmHg but not < 80 mmHg. The nitroglycerine was prepared by diluting a 10-mg/mL ampoule in 100 mL D5W (5% dextrose in water), and was administered using an infusion pump. In the nifedipine group, the content (100 µL) of a 10-mg capsule of nifedipine was deposited sublingually before induction of anesthesia, until the therapeutic goal had been reached. All stages of patient management before induction of anesthesia were performed under the supervision of an obstetrician.

The study solutions were prepared by an anesthesiologist who took no further part in the study. The anesthesiologists who evaluated the patients were not aware of their group allocation. All other individuals involved in the study, including the assisting nurses and the data analysts, were also blinded to the group allocations and the drugs administered throughout the trial. Patients were premedicated with 10 mg metoclopramide and 50 mg ranitidine intravenously, 30 min before induction of anesthesia. Monitors included pulse oximetry for oxygen saturation (SpO2); electrocardiogram (ECG); end-tidal carbon dioxide (EtCO2); and noninvasive automatic BP monitoring for systolic arterial pressure (SAP), diastolic arterial pressure (DAP), mean arterial pressure (MAP), and heart rate (HR).

After pre-oxygenation for 5 min, patients were given the study drugs. One hundred seconds after receiving the study drug, anesthesia was induced with 5 mg/kg thiopental sodium given over 10-15 s, and an assistant applied cricoid pressure from the time of induction of anesthesia until the airway was secured. Laryngoscopy and tracheal intubation were performed 60 s after giving 1. 5 mg/kg succinylcholine, thus ensuring that the time from administration of the study drug to tracheal intubation was 3 min. Anesthesia was maintained with 50% nitrous oxide in oxygen and 1. 2% isoflurane. Atracurium was used for muscle relaxation. EtCO2 was maintained at 33–40 mmHg throughout surgery.

After delivery of the baby, analgesia was provided by 0. 1 mg/kg morphine, and 20 IU IV Syntocinon in 1 L Ringer’s lactate was infused to initiate uterine contraction. HR, SBP, DBP and MBP, ECG in lead II, and SpO2 were simultaneously recorded in the mother at pre-induction, pre-intubation (2 min after giving test drug), and at 1, 3, 5, and 10 min after intubation. During the peri-intubation period, occurrence of any type of arrhythmia, or cardiovascular complications (such as hypo- or hypertension) was noted. Hypertension was defined as an increase in BP to more than 120% of baseline values, and when BP was reduced to less than 80% of the baseline, it was considered as hypotension. After completion of surgery, the residual effects of neuromuscular blockade were reversed with 0. 05 mg/kg neostigmine and 0. 02 mg/kg atropine. Apgar scores were evaluated at 1, 5, and 10 min. The person recording the data was blinded to the study drug administered. Statistical analysis was performed using SPSS version 16. 0. MBP was taken as DBP plus 1/3 × (SAP-DAP). Statistical comparisons among the groups were performed using 2-way analysis of variance (ANOVA), followed by an unpaired t-test with Bonferroni’s correction. Hemodynamic responses to induction and intubation in a given group were analyzed using a repeated-measurements ANOVA (1-way ANOVA) followed by a paired t-test with Bonferroni’s correction. Continuous variables are presented as mean ± SD. Ordinal variables are presented as numbers (%). The Chi-squared test was applied to categorical data with Yates’ correction. Alternatively, when any expected number was less than 5, Fisher’s exact method was used. P < 0. 05 was considered the minimum level of statistical significance.

## 4. Results

**Figure 1.**
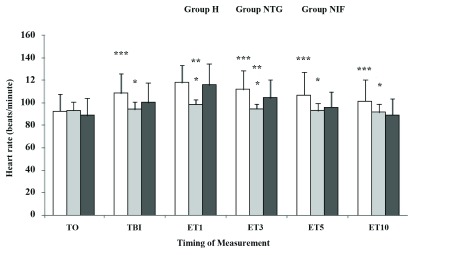
Heart Rate Changes After Different Time Intervals Following Intubation Data are presented as Mean ± SD. T0, baseline; TBI, just before intubation; ET1, 10 min after intubation. Group H, Hydralazine-treated group; Group NTG, Nitroglycerine-treated group; Group NIF, Nifedipine-treated group. *P < 0. 05 vs. Group NIF; **P < 0. 05 vs. Group H; ***P < 0. 05 vs. Group H.

Demographic characteristics, parity, baseline (preoperative) hemodynamic data, duration of laryngoscopy, and Cormack-Lehane grades of direct laryngoscopy were comparable between the 3 groups (Table 1). HR was significantly lower just before laryngoscopy and 1, 3, 5, and 10 min after endotracheal intubation in group NTG compared with group NIF (P < 0. 05) (Figure 1). HR was also significantly lower at 1 and 3 min after endotracheal intubation in group NTG compared with group H (P < 0. 05) (Figure 1). Patients in group NIF and group H showed a significant increase in HR just before laryngoscopy and 1, 3, 5, and 10 min after intubation, compared to baseline (P < 0. 05). The patients in group NTG showed no significant increases in HR after laryngoscopy and intubation.

**Table 1.** Maternal Demographic Data, Parity, Gestational Age, Baseline Hemodynamic Data, Duration of Laryngoscopy, and Cormack-Lehane Grades of Direct Laryngoscopy for Patients in the 3 Groups, (No significant difference in any parameters among groups)

	Group H ^a^ (n = 40)	Group NTG ^a^ (n = 40)	Group NIF ^a^ (n = 40)
Age, y, Mean ± SD	27. 0 ± 5. 1	28. 1 ± 4. 7	28. 7 ± 5. 3
Weight, kg, Mean ± SD	85. 7 ± 5. 6	84. 4 ± 5. 1	84. 9 ± 5. 1
Primipara/Multipara	28/12	25/15	27/13
Urgent/elective	32/8	35/5	33/7
HR ^a^, beats/min, Mean ± SD	89. 0 ± 15. 6	93. 3 ± 7. 6	92. 7 ± 15. 3
SAP ^a^, mmHg, Mean ± SD	169. 3 ± 11. 1	165. 3 ± 7. 8	167. 6 ± 11. 4
DAP ^a^, mmHg, Mean ± SD	109. 4 ± 8. 9	107. 4 ± 4. 2	108. 3 ± 8. 6
Mean arterial pressure, mmHg, Mean ± SD	129. 3 ± 8. 1	126. 7 ± 3. 8	128. 1 ± 7. 4
Duration of laryngoscopy, s, Mean ± SD	10. 0 ± 1. 7	09. 9 ± 1. 5	10. 5 ± 1. 5
C-L ^a^ grade of direct laryngoscopy, No.			
C-L 1	16	17	21
C-L 2	24	23	19
C-L 3	0	0	0

^a^ Abbreviations: C-L, Cormack-Lehane; DAP, Diastolic arterial pressure; Group H, Hydralazine-treated group; Group NTG, Nitroglycerine-treated group; Group NIF, Nifedipine-treated group; HR, Heart rate; SAP, Systolic arterial pressure

**Figure 2.**
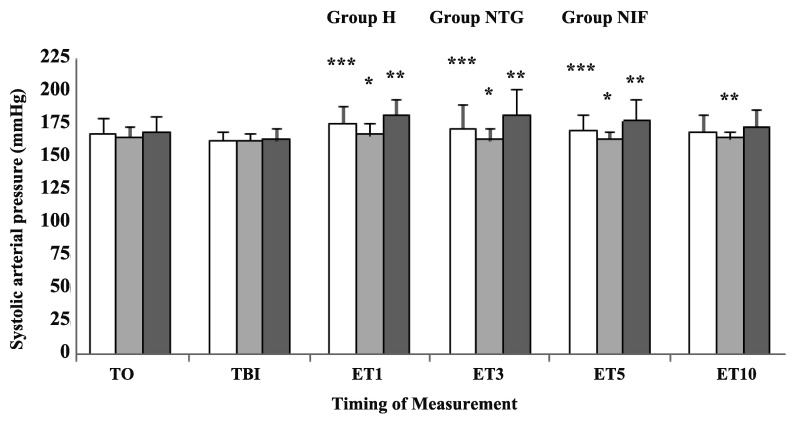
Systolic Arterial Blood Pressure Changes After Different Time Intervals Following Intubation Data are presented as Mean ± SD. T0, baseline; TBI, just before intubation; ET1, 10 min after intubation. Group H, Hydralazine-treated group; Group NTG, Nitroglycerine-treated group; Group NIF, Nifedipine-treated group. *P < 0. 05 vs. Group NIF; **P < 0. 05 vs. Group H; ***P < 0. 05 vs. Group H.

**Figure 3.**
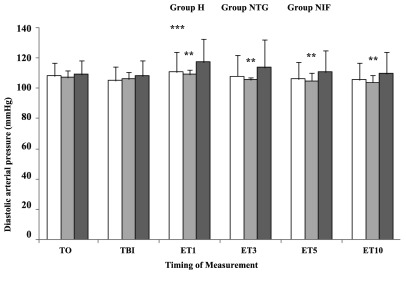
Diastolic Arterial Blood Pressure Changes After Different Time Intervals Following Intubation Data are presented as Mean ± SD. T0, baseline; TBI, just before intubation; ET1, 10 min after intubation. Group H, Hydralazine-treated group; Group NTG, Nitroglycerine-treated group; Group NIF, Nifedipine-treated group. **P < 0. 05 vs. Group H; ***P < 0. 05 vs. Group H.

SAP was significantly lower at 1, 3, and 5 min after endotracheal intubation in group NTG compared with group NIF (P < 0. 05) (Figure 2). In addition, SAP was significantly lower at 1, 3, 5, and 10 min after endotracheal intubation in group NTG compared with group H (P < 0. 05) (Figure 2). Patients in group H showed a significant increase in SAP just before laryngoscopy, and 1, 3, and 5 min after intubation, compared to baseline (P < 0. 05). Patients in group NIF showed a significant increase in SAP just before laryngoscopy, and 1 and 3 min after intubation, compared to baseline (P < 0. 05). The patients in group NTG showed no significant increases in SAP after laryngoscopy and intubation. DAP was significantly lower at 1, 3, 5, and 10 min after endotracheal intubation in group NTG compared with group H (P < 0. 05) (Figure 3). Patients in group H and NIF showed a significant increase in DAP at 1 min after intubation compared to baseline (P < 0. 05). The patients in group NTG showed no significant increases in DAP after laryngoscopy and intubation.

**Figure 4.**
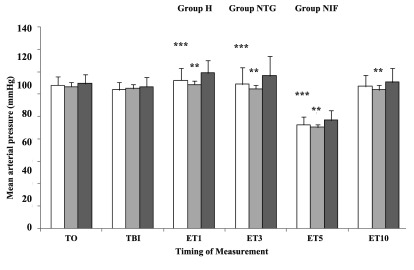
Mean Arterial Blood Pressure Changes After Different Time Intervals Following Intubation Data are presented as Mean ± SD. T0, baseline; TBI, just before intubation; ET1, 10 min after intubation. Group H, Hydralazine-treated group; Group NTG, Nitroglycerine-treated group; Group NIF, Nifedipine-treated group. **P < 0. 05 vs. Group H; ***P < 0. 05 vs. Group H.

MAP was significantly lower at 1, 3, and 10 min after endotracheal intubation in group NTG compared with group H (P < 0. 05) (Figure 4). Patients in group H showed a significant increase in MAP at 1, 3, and 5 min after intubation compared to baseline (P < 0. 05). Patients in group NIF showed a significant increase in MAP just before laryngoscopy, and 1 and 3 min after intubation, compared to baseline (P < 0. 05). The patients in group NTG showed no significant increases in MAP after laryngoscopy and intubation.

The incidence of hypotension was significantly greater in group NIF compared with group H or group NTG [15 (37. 5%) vs. 8 (20%) vs. 5 (12. 5%) respectively, P = 0. 025]. There were 2 patients in group H who developed arrhythmia, while no patients in group NIF or group NTG had arrhythmia (P > 0. 05). There was no significant difference between the 3 groups regarding gestational age, birth weight, and Apgar scores (Table 2). 

**Table 2.** Neonatal Data for the 3 Groups, (Significant differences among groups)

	Group H ^a^ (n = 40)	Group NTG ^a^ (n = 40)	Group NIF ^a^ (n = 40)
Gestational age, wk, Mean ± SD	36. 7 ± 0. 85	36. 4 ± 1. 1	36. 5 ± 1. 1
Birth weight, g, Mean ± SD	2873. 7 ± 403. 2	2766. 0 ± 514. 7	2706. 2 ± 241. 6
Apgar score < 7, No.			
1 min	6	4	5
5 min	0	0	0
10 min	0	0	0

^a^ Abbreviations: Group H, Hydralazine-treated group; Group NTG, Nitroglycerine-treated group; Group NIF, Nifedipine-treated group

## 5. Discussion

Our study showed that nitroglycerine, administered by continuous infusion during the peri-induction period of general anesthesia, was more effective than hydralazine or nifedipine in preventing increases in HR, SAP, DAP, and MAP after laryngoscopy and intubation, in patients with severe preeclampsia undergoing cesarean delivery.

Direct laryngoscopy and tracheal intubation cause an increase in BP and HR (20). This cardiovascular response is presumed to be a sympathetic reflex response to mechanical stimulation of the larynx and trachea. It involves an average increase in BP of 40–50%, and a 20% increase in HR (21). Significant elevations in serum levels of norepinephrine and epinephrine subsequent to the laryngoscopy, with and without tracheal intubation, have been reported (22-24). The pressor response to laryngoscopy and intubation increases myocardial oxygen requirement and risk of cerebrovascular accidents, decreases uterine blood flow, and induces cardiac arrhythmias and pulmonary edema (1, 2, 5, 25). Many drugs have been used for attenuation of these responses, but their relative efficacies have not been assessed (26, 27). Although lidocaine is commonly used for attenuation of the adrenergic response, its efficiency, particularly in severe preeclampsia, has been questioned (2). Preoperative opioids have been advocated for attenuation of the adrenergic response, and indeed short-acting opioids such as fentanyl have been used effectively at induction of anesthesia (28). However, opioids are avoided by some clinicians, as they may cause substantial respiratory depression in an already compromised fetus.

Our study showed that hydralazine was ineffective for attenuation of the stress caused by laryngoscopy during induction of general anesthesia. This may be due to the slow onset and variable duration of action of this drug, as well as compensatory tachycardia, which makes it difficult to titrate its action against the hypertensive response (29, 30). Hill et al. (31) showed that intranasal nitroglycerine can prevent an increase in BP following laryngoscopy and intubation. Van den Berg and colleagues (32) compared the effects of magnesium sulfate, esmolol, lidocaine, and nitroglycerine on the prevention of stress in laryngoscopy, and showed that nitroglycerine successfully prevented an increase in BP. On the contrary, magnesium sulfate and lidocaine did not have any effect on the hemodynamic changes following intubation. Further, in a study by Mikawak et al. (33), it was shown that administration of a single dose of intravenous nitroglycerine was a safe and effective method for attenuation of the hypertensive response following intubation. In a study performed in Greece (34, 35) women were enrolled to receive nitroglycerine before induction of anesthesia, and it was found that nitroglycerine effectively attenuated the increase in BP after laryngoscopy. Similarly, no increase in BP was seen in patients who received nitroglycerine prior to CABG surgery (35). These results are all in agreement with the success rates observed in the current study. The purpose of treating severe hypertension is to prevent the loss of cerebral autoregulation (causing encephalopathy and hemorrhage) and to avoid congestive heart failure. Generally, physicians aim to keep SBP between 140 and 160 mmHg and DBP between 90 and 110 mmHg, because at these BP levels a reduction in either uteroplacental blood flow or cerebral perfusion pressure is unlikely. Nitroglycerine appears to maintain SAP and DAP within the desired range more successfully than nifedipine or hydralazine.

Nifedipine is one of several agents that have been used for the reduction of BP in severe preeclampsia. As a calcium channel blocker, it causes peripheral arterial vasodilatation (36). Nifedipine was selected for analysis in this study as it is one of the most commonly-used drugs in the management of pregnancy-induced hypertension in developing countries (37). The route of administration of nifedipine used in the current study ensured immediate contact of the total drug dose with the sublingual mucosa, where it is mainly absorbed; nevertheless, we cannot rule out partial absorption throughout the gastrointestinal tract due to unintentional swallowing, and therefore, the combined effect of both events on absolute bioavailability is not known.

It has been shown that modes of administration of nifedipine that avoid first-pass metabolism in the gastrointestinal tract, such as use of a sublingual perforated capsule, result in greater bioavailability, and a faster accumulation and more stable concentration of nifedipine in serum than methods that do not avoid first-pass metabolism (oral administration of an aspirated capsule or chewable capsule) (38). In addition, sublingual administration methods allow for a more gradual decrease in BP than oral administration methods. Therefore, clinically, they are regarded as the most appropriate to achieve rapid onset and better control of the effects of nifedipine subsequent to its administration (38).

The finding that the nifedipine-treated group showed both greater MAP variability and a greater maternal HR response than the nitroglycerine-treated group after laryngoscopy and tracheal intubation implies that, under the present study conditions, sublingual nifedipine induces a greater variability in the arterial baroreflex system, through mechanisms that cannot be ascertained by the present study. A study by Fenakel et al. (39) indicated that nifedipine has greater efficacy than hydralazine in achieving control of BP in severe preeclampsia. In keeping with our results, Kwawukume and Ghosh, in their study on 114 severely preeclamptic patients, found that nifedipine was more effective in controlling BP than hydralazine (40). However, in the present study, nifedipine and hydralazine were less effective in controlling BP than nitroglycerine. Not only did the hypotensive effect of nitroglycerine begin earlier, but the therapeutic goal was also attained faster and with greater precision than with sublingual nifedipine or intravenous hydralazine. Maternal and fetal tolerance of intravenous nitroglycerine therapy appeared to be excellent in our study. Only 5 episodes of hypotension were observed in the NTG group; additionally, no case of tachycardia was seen. In the NIF and H groups, maternal hypotension was significantly more frequent; however, it was not associated with neonatal death. We noticed that a 1-min Apgar score of less than 7, seen in 10% of patients, appeared to be related to prematurity and maternal disease rather than adverse effects of the drug administered.

In conclusion, the present study found that, in women with severe preeclampsia who are managed with controlled extracellular volume expansion and MgSO4 loading and maintenance doses, a continuous IV infusion of nitroglycerine was able to attenuate the pressor response to laryngoscopy and intubation to a greater extent, faster, and more precisely than the use of sublingual nifedipine or IV hydralazine, without significant adverse effects on the baby. Limitations of our study are the lack of a proper control group and incomplete data on the potential adverse effects of hydralazine, nitroglycerine, or nifedipine on the fetus and newborn. Although more research must be done, we believe that nitroglycerine infusion may provide safe and effective prophylaxis for patients with severe preeclampsia undergoing cesarean delivery under general anesthesia, in attenuating hemodynamic responses to laryngoscopy and tracheal intubation.

## References

[1] Hodgkinson R, Husain FJ, Hayashi RH (1980). Systemic and pulmonary blood pressure during caesarean section in parturients with gestational hypertension.. Can Anaesth Soc J..

[2] Connell H, Dalgleish JG, Downing JW (1987). General anaesthesia in mothers with severe pre-eclampsia/eclampsia.. Br J Anaesth..

[3] Evans CS, Gooch L, Flotta D, Lykins D, Powers RW, Landsittel D (2011). Cardiovascular system during the postpartum state in women with a history of preeclampsia.. Hypertension..

[4] Kronborg CS, Gjedsted J, Vittinghus E, Hansen TK, Allen J, Knudsen UB (2011). Longitudinal measurement of cytokines in pre-eclamptic and normotensive pregnancies.. Acta Obstet Gynecol Scand..

[5] Lawes EG, Downing JW, Duncan PW, Bland B, Lavies N, Gane GA (1987). Fentanyl-droperidol supplementation of rapid sequence induction in the presence of severe pregnancy-induced and pregnancy-aggravated hypertension.. Br J Anaesth..

[6] Magee LA, Abalos E, von Dadelszen P, Sibai B, Easterling T, Walkinshaw S (2011). How to manage hypertension in pregnancy effectively.. Br J Clin Pharmacol..

[7] Ashton WB, James MF, Janicki P, Uys PC (1991). Attenuation of the pressor response to tracheal intubation by magnesium sulphate with and without alfentanil in hypertensive proteinuric patients undergoing caesarean section.. Br J Anaesth..

[8] Ramanathan J, Sibai BM, Mabie WC, Chauhan D, Ruiz AG (1988). The use of labetalol for attenuation of the hypertensive response to endotracheal intubation in preeclampsia.. Am J Obstet Gynecol..

[9] Strauss RG, Keefer JR, Burke T, Civetta JM (1980). Hemodynamic monitoring of cardiogenic pulmonary edema complicating toxemia of pregnancy.. Obstet Gynecol..

[10] Shinider SM, Levinson G (1987). Anesthesia for obstetrics.. Anesthesiology..

[11] Longmire S, Leduc L, Jones MM, Hawkins JL, Joyce TH, 3rd, Cotton DB (1991). The hemodynamic effects of intubation during nitroglycerin infusion in severe preeclampsia.. Am J Obstet Gynecol..

[12] Trapani A, Jr, Goncalves LF, Pires MM (2011). Transdermal Nitroglycerin In Patients With Severe Preeclampsia With Placental Insufficiency: Effect On Doppler Velocimetry Of The Uterine, Umbilical And Middle Cerebral Arteries.. Ultrasound Obstet Gynecol..

[13] Kumar N, Batra YK, Bala I, Gopalan S (1993). Nifedipine attenuates the hypertensive response to tracheal intubation in pregnancy-induced hypertension.. Can J Anaesth..

[14] Lenfant C (2001). Working group report on high blood pressure in pregnancy.. J Clin Hypertens (Greenwich, Conn)..

[15] Rey E, LeLorier J, Burgess E, Lange IR, Leduc L (1997). Report of the Canadian Hypertension Society Consensus Conference: 3. Pharmacologic treatment of hypertensive disorders in pregnancy.. CMAJ..

[16] Cutler JA (1998). Calcium-channel blockers for hypertension--uncertainty continues.. N Engl J Med..

[17] Grossman E, Messerli FH, Grodzicki T, Kowey P (1996). Should a moratorium be placed on sublingual nifedipine capsules given for hypertensive emergencies and pseudoemergencies?. JAMA..

[18] Cunningham FG, Williams JW, Leveno KJ, Bloom S, Hauth JC, Rouse DJ (1997). Williams obstetrics..

[19] Mabie WC, Gonzalez AR, Sibai BM, Amon E (1987). A comparative trial of labetalol and hydralazine in the acute management of severe hypertension complicating pregnancy.. Obstet Gynecol..

[20] Stoelting RK (1977). Circulatory changes during direct laryngoscopy and tracheal intubation: influence of duration of laryngoscopy with or without prior lidocaine.. Anesthesiology..

[21] Bruder N, Ortega D, Granthil C (1992). [Consequences and prevention methods of hemodynamic changes during laryngoscopy and intratracheal intubation].. Ann Fr Anesth Reanim..

[22] Russell WJ, Morris RG, Frewin DB, Drew SE (1981). Changes in plasma catecholamine concentrations during endotracheal intubation.. Br J Anaesth..

[23] Shribman AJ, Smith G, Achola KJ (1987). Cardiovascular and catecholamine responses to laryngoscopy with and without tracheal intubation.. Br J Anaesth..

[24] Park BY, Jeong CW, Jang EA, Kim SJ, Jeong ST, Shin MH (2011). Dose-related attenuation of cardiovascular responses to tracheal intubation by intravenous remifentanil bolus in severe pre-eclamptic patients undergoing Caesarean delivery.. Br J Anaesth..

[25] Miyake W, Oda Y, Ikeda Y, Tanaka K, Hagihira S, Iwaki H (2010). Effect of remifentanil on cardiovascular and bispectral index responses following the induction of anesthesia with midazolam and subsequent tracheal intubation.. J Anesth..

[26] Morison DH (1987). Anaesthesia and pre-eclampsia.. Can J Anaesth..

[27] Talebi H, Nourozi A, Fateh S, Mohammadzadeh A, Eghtesadi-Araghi P, Jabbari S (2010). Effects of oral clonidine premedication on haemodynamic response to laryngoscopy and tracheal intubation: a clinical trial.. Pak J Biol Sci..

[28] Rout CC, Rocke DA (1990). Effects of alfentanil and fentanyl on induction of anaesthesia in patients with severe pregnancy-induced hypertension.. Br J Anaesth..

[29] Ramanathan J (1992). Pathophysiology and anesthetic implications in preeclampsia.. Clin Obstet Gynecol..

[30] Ring G, Krames E, Shnider SM, Wallis KL, Levinson G (1977). Comparison of nitroprusside and hydralazine in hypertensive pregnant ewes.. Obstet Gynecol..

[31] Hill AB, Bowley CJ, Nahrwold ML, Knight PR, Kirsh MM, Denlinger JK (1981). Intranasal administration of nitroglycerin.. Anesthesiology..

[32] van den Berg AA, Savva D, Honjol NM (1997). Attenuation of the haemodynamic responses to noxious stimuli in patients undergoing cataract surgery. A comparison of magnesium sulphate, esmolol, lignocaine, nitroglycerine and placebo given i. v. with induction of anaesthesia.. Eur J Anaesthesiol..

[33] Mikawa K, Hasegawa M, Suzuki T, Maekawa N, Kaetsu H, Goto R (1992). Attenuation of hypertensive response to tracheal intubation with nitroglycerin.. J Clin Anesth..

[34] Fassoulaki A, Kaniaris P (1983). Intranasal administration of nitroglycerine attenuates the pressor response to laryngoscopy and intubation of the trachea.. Br J Anaesth..

[35] Mahajan RP, Ramachandran R, Saxena N (1993). Topical nitroglycerin prevents the pressor response to tracheal intubation and sternotomy in patients undergoing coronary artery bypass graft surgery.. Anaesthesia..

[36] Ferlinz J (1986). Drugs Five Years Later Nifedipine in Myocardial Ischemia, Systemic Hypertension, and Other Cardiovascular Disorders.. Ann Intern Med..

[37] Jegasothy R, Paranthaman S (1996). Sublingual nifedipine compared with intravenous hydrallazine in the acute treatment of severe hypertension in pregnancy: potential for use in rural practice.. J Obstet Gynaecol Res..

[38] Kubota R, Komiyama T, Shimada H (2001). Evaluation of the method for nifedipine administration for a rapid onset of clinical effect: a clinical study in normal volunteers.. Yakugaku Zasshi..

[39] Fenakel K, Fenakel G, Appelman Z, Lurie S, Katz Z, Shoham Z (1991). Nifedipine in the treatment of severe preeclampsia.. Obstet Gynecol..

[40] Kwawukume EY, Ghosh TS (1995). Oral nifedipine therapy in the management of severe preeclampsia.. Int J Gynaecol Obstet..

